# A Cage Is a Cage, Unless You Educate. Rhetoric Negatively Impacts Support for a Novel Housing System for Laying Hens Unless the Public Are Educated

**DOI:** 10.3389/fvets.2022.797911

**Published:** 2022-02-18

**Authors:** Huw R. J. Nolan, Lauren M. Hemsworth, Jennifer A. Power-Geary, Peta S. Taylor

**Affiliations:** ^1^Animal Science, School of Environmental and Rural Science, Faculty of Science, Agriculture, Business and Law, University of New England, Armidale, NSW, Australia; ^2^Animal Welfare Science Centre, Faculty of Veterinary and Agricultural Sciences, University of Melbourne, Parkville, VIC, Australia

**Keywords:** furnished cage, animal welfare, perception, science, knowledge - attitude - behavior, language, free-range, eggs

## Abstract

It has been proposed that terminology on commercially available eggs can impact the manner in which the eggs are discussed and ultimately consumer support. In this paper we tested if the label of ‘furnished cage’ eggs is a barrier for its support in Australia. Furthermore, we examined if educational interventions could change support and the way furnished cages were discussed. Survey participants (*n* = 1,157) were recruited by a stratified random sample of Australian adults. The participants were surveyed on their demographics, attitudes toward the poultry industry and animal welfare, and their egg buying behavior. Participants were randomly assigned to one of four treatment groups; two control groups and two educational groups. Participants were shown one of three videos, the control groups were shown a video with general information about chickens, the educated groups were shown one of two almost identical videos that educated them on aspects of the egg-laying industry in Australia, and the welfare implications of different housing systems including furnished systems. The only difference between the two educational videos was the name given to the furnished housing system; one group was introduced to furnished cages, the other was introduced to furnished coops. Educated participants were more likely to support furnished eggs and discuss them more positively than the control groups. When asked to discuss their support for furnished systems, control group participants exposed to the term *cage* were more likely to discuss the impacts of caged environments than the other treatment groups. The study suggests any negative impacts of housing system terminology can be mitigated through educational interventions.

## Introduction

Animal welfare scientists investigate and promote new and improved methods to manage captive animals with the aim to improve animal welfare. Nevertheless, scientific findings are often at variance with the widely held beliefs of the general public (1–3). This may be partially because the scientific findings are not appropriately communicated to the general public; scientific information is often published in pay-walled journal articles and obscured behind confusing statistics and discipline-specific jargon. As such, the science rarely reaches the public in a form that might alter their perceptions. Furthermore, how consumers understand scientific information and form opinions affecting their choices as consumers is mediated by factors such as their socio-economic status, level of education, access to scientific findings and their understanding of key terminology gained from advertising and information (the rhetoric) (4). A large portion of the Australian public (71%) regard farm animal welfare to be of concern, particularly poultry welfare (5). Australians frequently perceive the welfare of laying hens to be poorer than other livestock species (6). Yet knowledge of farming practices have been reported to be low (7), education interventions may be a critical component of improving attitudes toward industry practices. The impact of education on industry support is evident by Erian and Phillips (8) that have shown that knowledge of production practices was positively associated with chicken meat consumption, suggesting that greater knowledge of industry practices results in increased consumer support.

Choices made when purchasing chicken eggs are also dependent on the public's perceptions and an understanding of the welfare issues associated with the systems used to house the laying hens, e.g., conventional cages vs. free-range housing (9). Leveraging the market is an effective mechanism for improving the practices that mitigate welfare issues (10). Such practices will only be sustained through preferences of egg-purchasers; when consumers exhibit a lower preference for eggs from novel housing systems, industry stakeholders are disincentivized from making changes and the status quo is maintained. Currently, consumers generally have negative perceptions of caged housing systems and more positive perceptions of free-range systems (11–13). This is reflected in the increase in sales of eggs from free-range housing (14). Australian free-range grocery egg sales continue to grow with free-range sales holding 57% of the grocery market value, followed by caged eggs at 30%, barn-laid eggs at 10% and specialty eggs at 3%, as reported in the 2019–2020 financial year (15). Scrinis et al. (16) coined the term *animal housing reductionism* to refer to the tendency for public discourse of animal welfare, ethics, language and labeling to be reduced to issues of housing.

Commercially, most eggs are marketed with names derived from how the hens are housed (e.g. *free-range, barn raised, caged*) which can act as branding devices signaling both food attributes and social rhetoric (17). The reasons why people purchase eggs from hens housed in different conditions are varied and in addition to the aforementioned factors, are also influenced by price, perceived quality, taste, societal expectations, environmental factors, as well as hen welfare (13, 18). Heuristics are the mental shortcuts to make quick judgements (19). For consumers, the terms that define eggs come with heuristics associated with a set of issues and values. The role of language on social issues such as race, gender, class has been investigated [see (20)] yet few studies have investigated the role of language on animal welfare related issues, such as consumer perceptions and buying behavior. Confinement has become a key issue in animal welfare discussions (21). Because of this, when consumers think of a cage, they may think of metal bars, confinement, and the occupant's reduced ability to express natural behaviors; they do not consider the positive factors such as reduced risk of hen-to-hen aggression or disease control. The heuristics that guide consumers to choose eggs from different housing systems are sometimes flawed, which is to be expected if the publics' scientific knowledge of housing systems is sparse. However, consumers have demonstrated a desire for improved welfare standards, and for information of these standards to be understandable quickly and transparently on animal products and packaging (22). Consumers will use the labeling as a proxy for welfare conditions (18) despite evidence that consumer knowledge of the meaning behind egg labels is low (23, 24). When presented with a novel egg type, whose name is derived from its housing system absent of further description or explanation, consumers may respond according to the heuristics associated with the system's name alone and, as these names have different meanings for different people, the levels of support will differ. Understanding the impact of terms used in egg labeling is important to understanding the future of hen welfare.

Conventional cage systems are in global decline; many countries are either phasing them out or have banned them altogether (25), replacing them with free-range and other cage-free systems. The changes are often being made in the belief that they will improve layer hen welfare (11, 12, 16) even though research has consistently illuminated negative welfare consequences of cage-free housing alternatives [see for instance (26–28)]. By way of a compromise, furnished cages were developed to minimize the negative welfare impacts of both cage and cage-free systems. Despite scientific research demonstrating the welfare benefits of furnished cages, there is no current market for furnished cage eggs in Australia. A reason for this might simply be the negative connotation associated with the descriptor “cage” (29). As pointed out by Weary et al. (3), consumers who want cage-free eggs will be less likely to support any type of caged housing system, even if *enriched* or *furnished*. As it is largely the egg-purchasers who drive change, if support from the public cannot be demonstrated, it is unlikely a furnished cage industry in Australia would be viable. Rohlf et al. (30) investigated the efficacy of online forums as a tool to educate the public about furnished cages with the aim to increase support. After educating, support for the introduction of furnished cages in Australia increased from 55 to 65% (30). The Rohlf et al. (30) pilot study provides some evidence that education can improve acceptance of a novel housing system by the Australian community, however the impact of the industry's involvement in their education process (contrasted by independent science-based education campaigns) and the language used during the education process was not assessed both of which may have contributed to the marginal increase in support.

We aimed to compare the effect of rhetoric and education on willingness to support a novel housing system for laying hens. Our hypotheses were threefold. Firstly, we hypothesized that the rhetoric used in naming egg types affects consumers' preferences for eggs from different housing systems. Second, we hypothesized that consumers' preferences for eggs from these housing systems will change when they are made aware of scientific knowledge about housing systems. Finally, we hypothesized that after being educated on current scientific thinking, discourse around hen housing would include more of the themes expressed in the education.

## Materials and Methods

### Survey Design

A subsample of the Australian public was surveyed regarding their attitudes toward hen welfare, housing and management in Australia. Specifically, the survey was designed to compare self-reported buying behavior when participants were introduced to a novel housing system, either with or without an educational intervention video and using different terminology, *Furnished Cage* or *Furnished Coop*.

The survey was informed by industry and community focus groups [six and seven participants, respectively; see (31) for information on focus groups]. In short, focus group discussions were used to help design knowledge questions of knowledge and values. Specifically, the use of furnished cage housing in the current study was chosen as none of the participants in the community focus group (*n* = 7) had heard of furnished cages, and further provided evidence of negative rhetoric with the use of the term *cage*. The term *coop* was chosen as it is synonymous with cage and even has similar connotations of confinement, “to be cooped up.” However, it is largely associated with backyard chicken keeping and not associated with industrial egg production. Therefore, we assumed it would not carry the same negative connotations as *cage*. The questions used in this study were part of a larger survey with many questions omitted from this paper (Supplementary Material).

### Sample Population and Recruitment

Recruitment and completion of the survey was through a paid online survey platform, Qualtrics XM (Provo, UT, USA). Recruitment of the participants was by a stratified random sample of adults in Australia. Set quotas were enforced to ensure participants were representative of the overall Australian population in relation to gender, age, and geographical location [(32); Table 1]. Responses were considered valid if the participant completed all sections, including open text responses and did not demonstrate *flatlining* i.e., the same response across multiple questions.

**Table 1:** Stratified random sampling of demographics of survey participants: original quotas (32) and valid percentage of survey responses.

**Demographic**	**Options**	**Survey**	**Survey**	**Original**
		**(count)**	**(%)**	**quota (%)**
Gender	Female	586	50.6	48
	Male	571	49.4	47
	Non-binary	-	-	5.0
Age	18–24	150	13.0	15.7
	25–34	201	17.4	17.7
	35–44	201	17.4	16.5
	45–54	193	16.7	16.3
	55–65	176	15.2	14.5
	65+	236	20.4	19.3
State	Australian Capital Territory	23	2.0	2.0
	New South Wales	375	32.4	32
	Northern Territory	6	0.5	1.0
	Queensland	232	20.1	20
	South Australia	82	7.1	7.0
	Tasmania	24	2.1	2.0
	Victoria	285	24.6	25.0
	Western Australia	130	11.2	11

### Experimental Design

**Figure 1:**
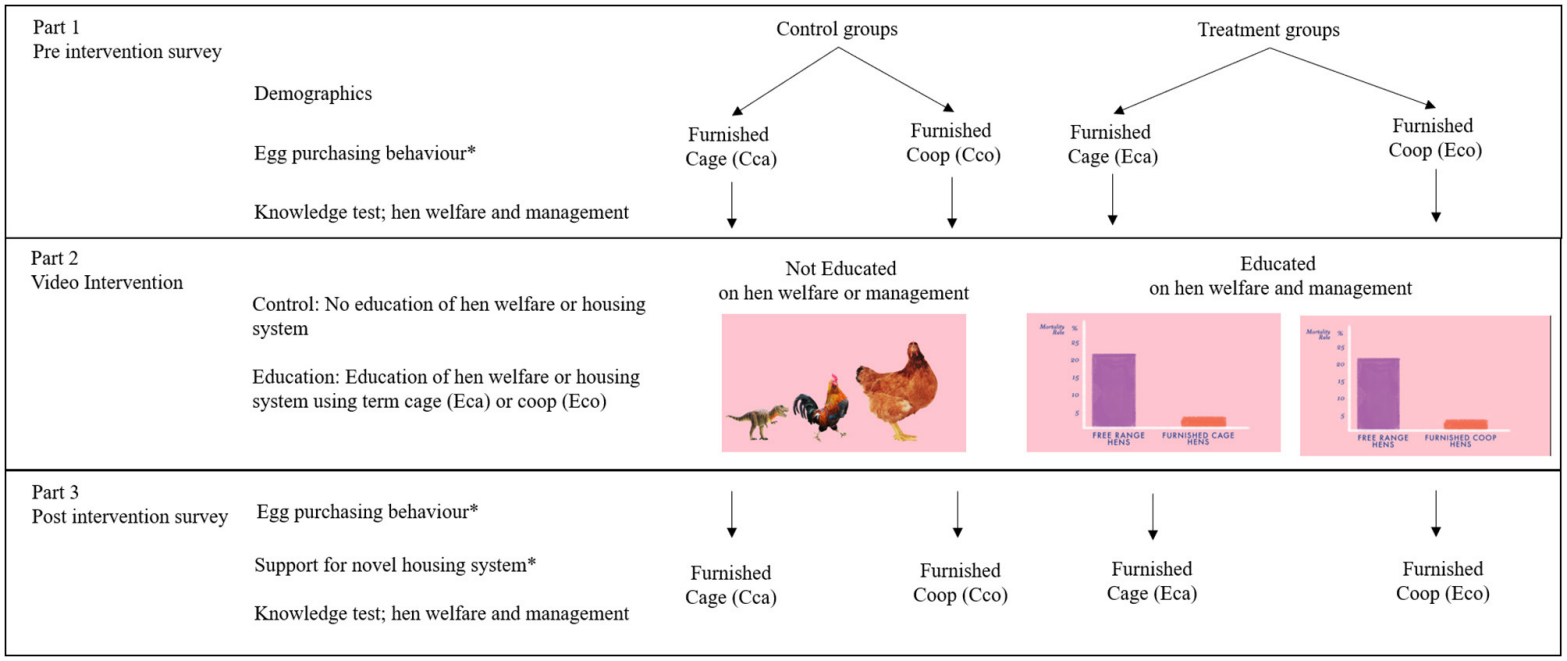
Visual representation of experimental methods and survey flow. Participants were randomly assigned to treatment groups and were asked a series of questions including their perspective and knowledge of hen welfare and the Australian egg industry (Part 1). Participants were then shown either a control video or education video that discussed general chicken facts or specific facts regarding hen welfare and housing systems respectively (Part 2 intervention). Post video intervention participants were asked the same questions as part 1 and if they would support furnished cage/coop housing in Australia (Part 3). * indicates that the questions asked to participants differed in the language used between treatment groups; furnished cage (Cca and Eca) or furnished coop (Cco and Eco).

At the commencement of the survey, using Qualtrics' randomization function, the participants were randomly assigned to one of four treatment groups; two control groups and two educated groups. Within the treatment groups participants were exposed to either the term Furnished Cage (Cca and Eca) or Furnished Coop (Cco and Eco). Participants were shown an educational intervention video (Eca and Eco) or a control video (Cca and Cco). At no point were Cca and Eca exposed to the term Furnished Coop and vice versa (Figure 1).

### Pre-video Intervention

Part 1 of the survey included questions regarding demographics (*n* = 9), egg-choice preferences (*n* = 5) and hen welfare (*n* = 5). Additionally, knowledge of hen welfare and management practices were assessed with true or false statements (*n* = 9), and open text questions (*n* = 4) where participants were asked to define four terms associated with hen welfare and egg production. For all knowledge questions, participants were able to answer *I don't know*. Participants were then shown one of three videos dependent on the participants allocated treatment group (Figure 1).

### Video Intervention

Participants in the education groups (Eca and Eco) were provided with a 3 min 43 s video containing science-based information about various aspects of hen welfare and commercial hen housing. Scripts for the video narration were informed by current scientific information and consultation with poultry industry representatives (Supplementary Material). Both education videos were identical except when introducing the furnished cage housing system. From this point, the narrator either said “furnished cage” (Eca) or “furnished coop” (Eco) but the descriptions of both systems were otherwise identical. Conventional cage and free-range housing were also discussed. Both housing systems were presented with even amounts of positive and negative information regarding hen welfare. Information about housing systems were presented using high modality language only if scientific evidence supported the assertion in all contexts e.g., in furnished systems, hens are housed in groups of more natural group size than hens in non-caged systems (33). When there was doubt, or the truth of the statement depended on the context, low modality language was used, e.g., Conventional caged system: *At the end of their production life hens are more likely to have osteoporosis and bone fractures*. Free-range system: *Also, hens are more likely to collide with objects and each other which can cause injuries*.

Participants in the control groups (Cca and Cco) were provided with a 3 min 32 second video containing general information regarding chickens (Supplementary Material). The video contained statements such as “Vocalizations are an important communication tool for chickens,” “There have been around 30 different [chicken] vocalizations described although we still don't know what many of them mean” (34), and “After hatching, chicks stay close to the mother hen for protection and to gain some valuable lessons, such as what is good to eat and what is potentially harmful” (35). The video did not contain information related to hen welfare, the egg industry, or answers to any of the knowledge questions previously asked in part 1.

All videos were professionally animated in the same style and narrated by the same person. The videos were produced to be clear and straightforward; the narration was friendly and non-authoritarian.

Although participants in control groups were not educated on furnished housing systems, the survey contained questions related to their perceptions of furnished cage housing systems. Therefore, two control groups were necessary to assess the impact of rhetoric. As such, Cca and Eca were asked about Furnished *cages*, Cco and Eco were asked about Furnished *coops*.

### Post-video Intervention

After the video intervention (Part 3) participants were asked the questions from Part 1 again regarding their attitudes toward, and knowledge of, hen welfare and housing systems in Australia. Additionally, participants were asked if they *would support furnished cage/coop eggs in Australia* (responses: yes, no, maybe and yes but, followed by an open text option). Then all participants were invited to add an extra comment about furnished systems. In the form of an open text question, *please provide further comment on your answer above (e.g., why, why not, only if)*.

### Statistical Analysis

Data analysis was performed in SPSS statistical software (v22, IBM Crop, Armonk, NY, USA).

#### Pre-video Intervention

To ensure potential socio-demographic or value-based biases were not present between treatment groups prior to the treatment interventions, we compared support scores for each housing system, level of concern for hen welfare, perceptions of hen welfare, and the proportion of participants that did not know what furnished housing was between treatment groups with Chi-squared tests for independence.

The answers of the nine *True/False* knowledge questions were marked as either correct or incorrect. Responses of *I don't know* were marked as incorrect and the proportion of *I don't know* responses recorded. Open-text knowledge responses were marked correct or incorrect by two independent markers using an agreed upon set of criteria. As with the True/False questions, all responses of *I don't know/unknown*, etc. were marked as incorrect and proportions recorded. All marking was conducted blind, so neither the respondents nor the response treatments were known to the markers. One marker assessed all responses for a question while the other marker assessed a randomly selected 10% of the responses. Inter-marker reliability was analyzed using Cohen's kappa coefficients and were >0.80. The pre-video education knowledge scores from part 1 were analyzed between treatment groups using a Kruskal-Wallis test.

#### Post-video Intervention

Responses to the post-video intervention questions *Would you support Furnished Cage/Coop in Australia?* (response: yes, no, maybe, yes but) and *Which of the following hen housing systems would you consider buying from*? were analyzed using Chi-squared tests for independence. Z-tests were utilized for between treatment comparisons. Eca and Eco respondents were educated on the furnished systems, conventional cages and free-range housing. Therefore, it is reasonable to assume the proportion of the response choice *I am unfamiliar with this housing system*, would also change. However, as this was not the focus of the study, the *I am unfamiliar*... responses were removed from analysis to mitigate confounding results.

Open-text responses to the question “*Please provide further comment on your answer (e.g., why, why not, only if)*”, which was asked immediately after “*Would you support Furnished Cage/Coop in Australia”* were coded into 13 categories based on the themes discussed; s*eems good, seems bad, price, welfare, prefer free-range, I don't know what a Furnished Cage/Coop is, quality and taste, cage is a cage, gibberish* or *no comment, not sure/undecided*, and *other*. The categories were not mutually exclusive, as such individual responses could be classified into more than one category. Open text responses were blind coded by two authors. As above, one author marked all responses for a question while the other marked a randomly selected 10% of the responses. The two codes were compared using Cohen's kappa coefficients and was > 0.80. Categories were then analyzed between treatment groups using Chi-squared tests for independence. Z-tests were used for between treatment comparisons.

All *post-hoc* analyses were corrected for multiple comparisons using adjusted *p* values with the Bonferroni method.

This study was approved by the University of New England Human Ethics Committee (HE18–284).

## Results

We received 1,157 valid survey responses. There were no valid responses from participants identifying as a gender other than female or male and so quotas were relaxed and gender was represented by 50.6% female and 49.4% male. All participants were within 3% of our set quotas, with most being <1% (Table 1). Sample sizes for each of the four treatment groups were; two control groups (Cca, *n* = 285; Cco, *n* = 259) and for the two educated groups (Eca, *n* = 341; Eco, *n* = 272).

### Pre-video Intervention

Prior to the video intervention there was no difference between treatment groups in support for or opinion of animal welfare (Table 2). The Australian public demonstrated a high level of regard for animal welfare; most respondents (72%) thought hen welfare was either *very important* or *extremely important*, <1% or respondents did not consider hen welfare important at all. The number of responses of *not important at all* was below five in all treatment groups so the response was excluded from analysis.

**Table 2:** Pre-video intervention responses to survey questions before participants were shown either a control video using the term cage (control cage) or coop (control coop) or an educational video using the term cage (educated cage) or coop (educated coop).

**Question**	**Response**	**Cca**	**Cco**	**Eca**	**Eco**	**χ^2^**	***df***	***p***
Hen welfare is	Slightly important	3.8	5.1	5.8	5.1	5.78	12	0.76
	Moderately important	24.6	23.7	21.5	19.5			
	Very important	42.2	44.0	41.6	41.5			
	Extremely Important	29.5	27.2	31.0	33.8			
I think the welfare of Australian commercial hens is	Very bad	1.8	1.5	5.0	4.4	17.94	15	0.27
	Bad	10.2	10.0	11.7	11.8			
	Adequate	24.6	20.1	19.4	19.5			
	OK, but room for improvement	41.8	45.6	44.6	48.5			
	Good	19.6	20.5	16.7	13.6			
	Excellent	2.1	2.3	2.6	2.2			
Which of the following hen housing systems would you consider buying from? Furnished cage/coop	Never	53.5	44.9	43.4	42.8	11.46	12	0.49
	Rarely	18.4	21.2	19.8	18.2			
	Sometimes	15.2	18.6	18.7	23.3			
	Often	7.8	10.9	14.3	10.1			
	Always	5.1	4.5	3.8	5.7			
	Unknown	38.0	39.8	34.1	41.5	3.58	3	0.31
Which of the following hen housing systems would you consider buying from? Cage	Never	34.1	29.2	38.0	35.1	9.28	12	0.67
	Rarely	18.6	19.4	13.7	17.9			
	Sometimes	18.9	22.5	18.1	17.9			
	Often	15.6	18.2	17.7	17.6			
	Always	12.9	10.7	12.5	11.5			
	Unknown	4.6	2.3	1.8	3.7	4.82	3	0.20
Which of the following hen housing systems would you consider buying from? Free-range	Never	4.6	4.3	4.0	3.4	8.15	12	0.77
	Rarely	7.8	6.7	8.4	4.1			
	Sometimes	17.9	21.7	22.3	21.4			
	Often	29.5	29.5	29.3	29.3			
	Always	40.2	37.8	35.9	41.7			
	Unknown	1.1	1.9	1.1	2.2			
Which of the following hen housing systems would you consider buying from? Barn	Never	24.6	20.9	17.6	17.1	18.00	12	0.12
	Rarely	12.6	14.1	19.2	16.3			
	Sometimes	35.3	37.2	32.9	38.9			
	Often	18.0	22.2	24.3	19.4			
	Always	9.5	5.6	5.9	8.3			
	Unknown	9.4	9.7	7.6	7.4	1.59	3	0.67
Which of the following hen housing systems would you consider buying from? Aviary	Never	56.2	50.3	48.2	49.7	9.30	12	0.68
	Rarely	16.3	22.6	17.9	19.0			
	Sometimes	14.8	14.8	19.0	17.8			
	Often	7.9	9.7	11.3	7.4			
	Always	4.9	2.6	3.6	6.1			
	Unknown	42.0	40.2	39.1	40.1	0.57	6	0.90

Support for different housing systems did not differ between treatment groups, nor was there a significant difference in the number of participants that had previous knowledge of housing systems between treatment groups (Table 2). Furthermore, there was no difference in knowledge scores between treatment groups (20.3 ± 3.6 % correct; knowledge questions summarized in Table 3).

**Table 3:** Percentage of correct, incorrect and I don't know responses to True/False questions and open text questions.

	**Question**	**Correct**	**Incorrect**	**I don't know**
True/False	Free-range flocks consist of <5,000 hens	12.3	25.4	62.3
	Commercial strains of hens each produce over 300 eggs per year	35.4	5.2	59.4
	The current outdoor range stocking density for hens in free-ranged egg production systems is 10,000 hens/hectare	25.3	7.3	67.3
	Hens in free-range housing systems have no welfare problems	43.0	18.1	39.0
	Shed lights are on 24 h a day so each hen produces two eggs every day	14.5	28.2	57.3
	Yolk color is related to housing system	26.4	28.5	45.0
	Hens are killed between 16 and 25 weeks of age because their egg production decreases	15.0	21.3	63.7
	Chicken meat and eggs come from two different types of chickens	42.5	17.7	39.8
	Molting is practiced in Australia	17.8	7.0	75.2
Open Text	Define Free-range	11.5	76.2	12.3
	Define Beak-trimming	11.8	38.8	49.4
	Define Molting	4.0	30.3	65.7
	Define Feed Conversion Ratio	7.6	14.7	77.7

*Incorrect and I don't know were combined for analysis*.

### Post-video Intervention

#### Would You Support Furnished Cage/Coop in Australia?

**Figure 2:**
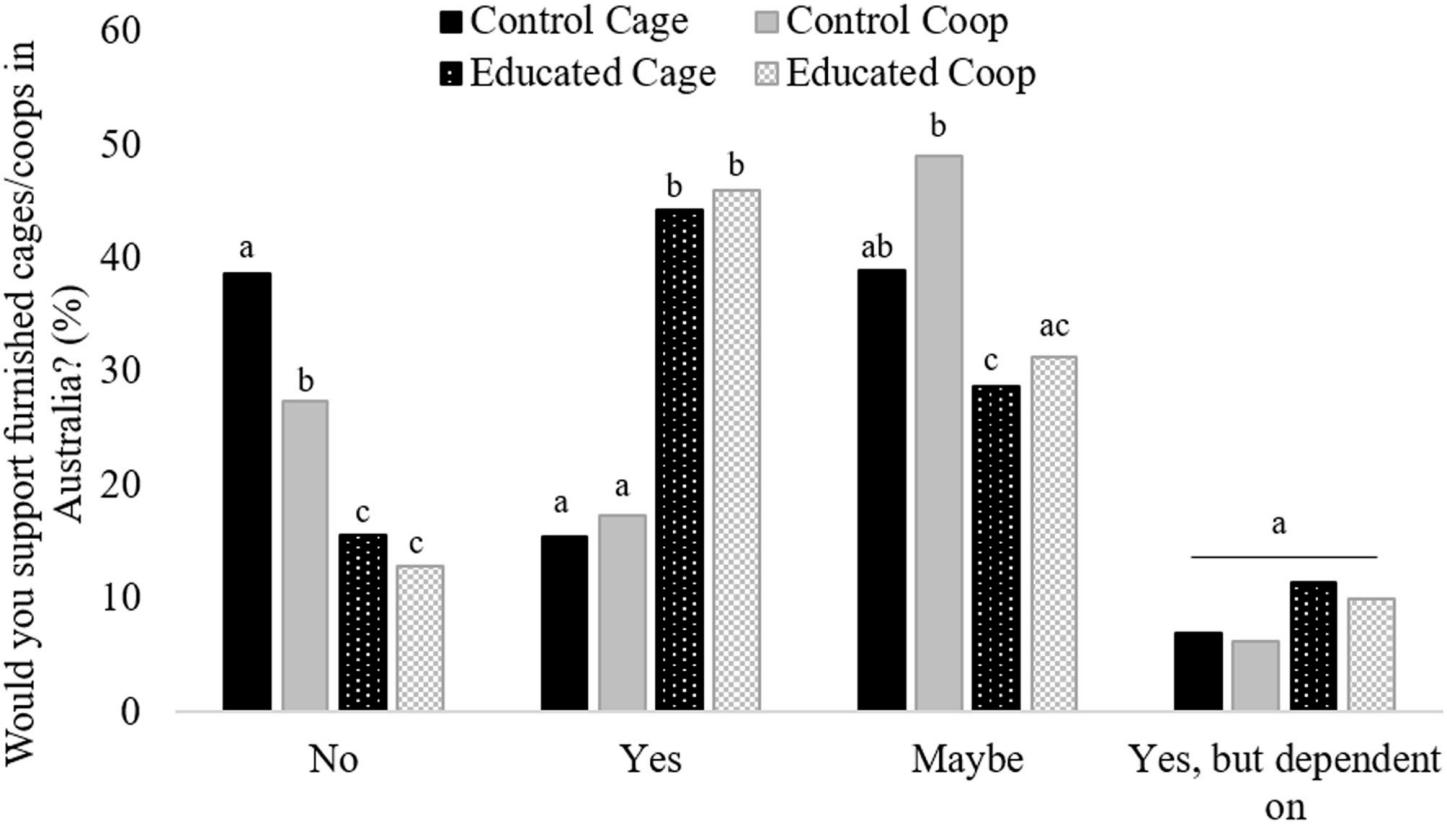
Frequency of response to the question “Would you support furnished cage/coop eggs in Australia?” from participants after they were shown either a control video using the term cage (control cage; solid dark gray bars) or coop (control coop; solid light gray bars) or an educational video using the term cage (educated cage; dark spotted bars) or coop (educated coop; gray checkered bars). Differing subscript indicates a significant difference between treatment groups for each response (*p* < 0.05).

Educated treatment groups were more likely to respond *Yes* when asked if they would support furnished cage/coop in Australia, than either control groups (χ^2^ = 153.0, *df* 9, *p* < 0.001; Figure 2). Control coop participants were less likely to respond *No* than Control cage and more likely to respond *Maybe* than either of the educated groups. Control coop participants were more likely to respond *Maybe* than Educated cage participants (Figure 2).

**Figure 3:**
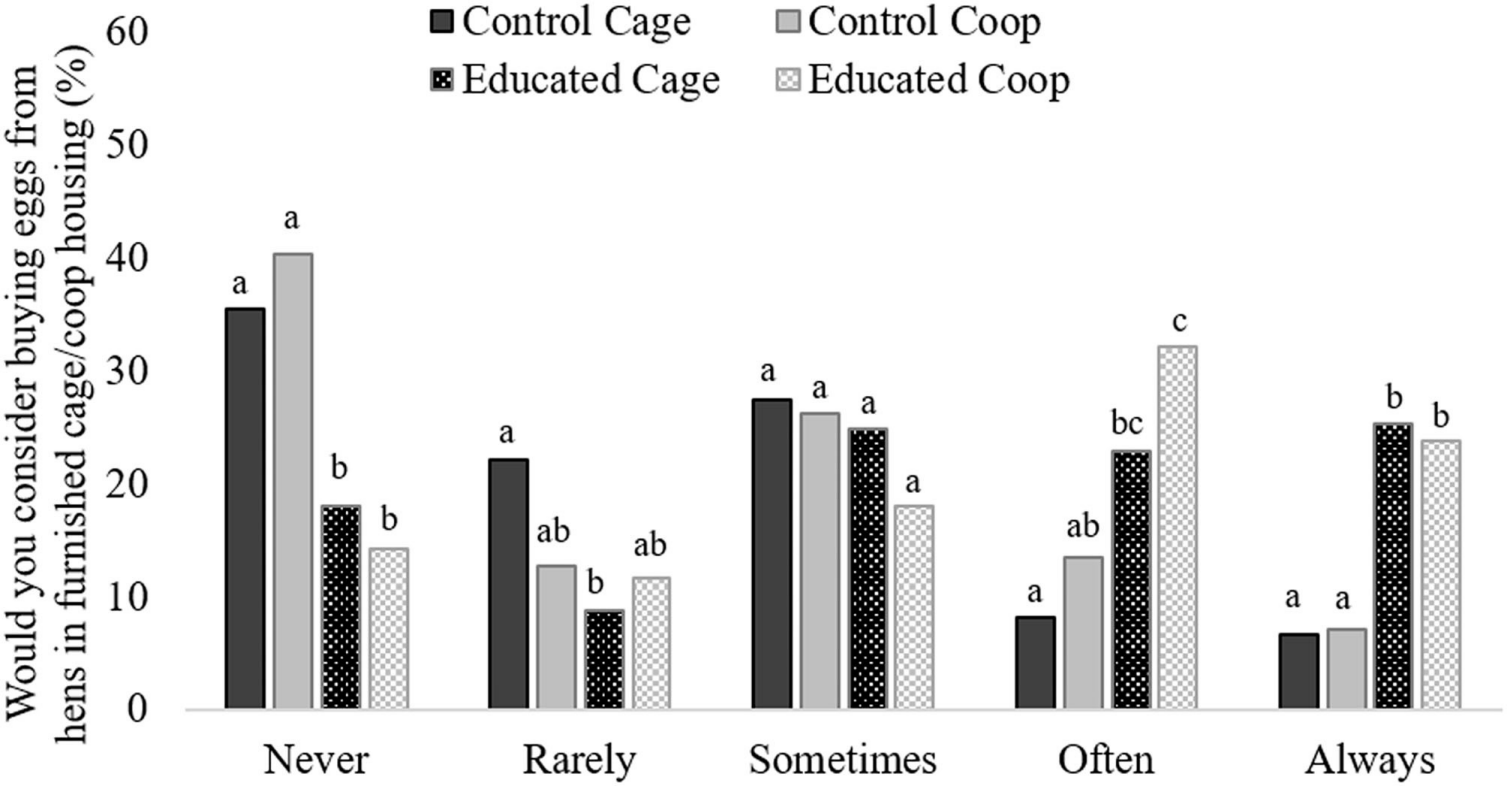
Frequency of response to the question “Which of the following hen housing systems would you consider buying from…furnished cage/coop?” from participants after they were shown either a control video using the term cage (control cage; solid dark gray bars) or coop (control coop; solid light gray bars) or an educational video using the term cage (educated cage; dark spotted bars) or coop (educated coop; gray checkered bars). Differing subscript indicates a significant difference between treatment groups for each response (*p* < 0.05).

The educated groups were less likely to respond *Never* and more likely to respond *Always* when asked if they would consider purchasing eggs from furnished cages/coops than the control groups (χ^2^ = 140.6, *df* 12, *p* < 0.001; Figure 3, Table 4). The control cage group were more likely to respond *Rarely* than Educated cage. Educated cage was more likely to respond *Often* than either control group. The educated cage group were more likely to respond *Often* than control cage (Figure 3, Table 4).

**Table 4:** Frequency of response to the question “Which of the following hen housing systems would you consider buying from?” from participants after they were shown either a control video using the term cage (control cage) or coop (control coop) or an educational video using the term cage (educated cage) or coop (educated coop).

**Which of the following hen housing systems would you consider buying from?**	**Response**	**Cca**	**Cco**	**Eca**	**Eco**	**χ^2^**	***df***	***p***
Furnished cage/coop	Never	36.2	36.6	13.1	8.8	140.55	12	<0.001
	Rarely	16.7	13.0	9.2	8.8			
	Sometimes	25.2	26.1	30.4	22.3			
	Often	10.2	15.5	26.2	35.9			
	Always	11.8	8.7	21.2	24.3			
Cage	Never	39.4	32.3	35.3	34.5	9.08	12	0.70
	Rarely	15.0	20.9	18.8	21.0			
	Sometimes	21.2	21.7	24.3	23.2			
	Often	14.4	14.2	13.6	13.5			
	Always	10.0	11.0	8.1	7.9			
Free-range	Never	5.0	5.5	4.0	3.8	42.63	12	<0.001
	Rarely	6.4	5.1	6.2	5.6			
	Sometimes	21.3	16.8	31.9	25.9			
	Often	25.9	27.0	31.5	36.1			
	Always	41.4	45.7	26.4	28.6			
Barn	Never	18.9	20.3	16.1	11.7	15.34	12	0.22
	Rarely	15.2	15.3	15.3	14.1			
	Sometimes	35.1	30.5	40.6	37.5			
	Often	21.6	25.8	20.7	28.1			
	Always	9.1	8.1	7.3	8.6			
Aviary	Never	42.7	47.1	35.6	33.8	17.23	12	0.14
	Rarely	19.0	13.4	20.9	18.1			
	Sometimes	23.7	22.9	30.1	24.4			
	Often	10.0	10.8	8.0	13.1			
	Always	4.7	5.7	5.5	10.6			

When asked to provide further comment as to why or why not participants would consider buying furnished cage/coop eggs, control groups were more likely to respond “*I don't know what that is”* (χ^2^ = 150.9, *df* 3, *p* < 0.001), “*I prefer free-range” (*χ^2^ = 7.7, *df* 3, *p* = 0.05), “*it seems good” (*χ^2^ = 125.3, *df* 3, *p* < 0.001) and mentioned “*welfare” (*χ^2^ = 57.6, *df* 3, *p* < 0.001). Furthermore, control participants that were asked about furnished cage were more likely to response “*seems bad” (*χ^2^ = 27.3, *df* 3, *p* < 0.001) and “*a cage is a cage” (*χ^2^ = 34.4, *df* 3, *p* < 0.001) than the control group that were asked about furnished coops and both educated groups (Table 5). There was no difference between treatment groups in the proportion of responses of “*Price,” “Quality and taste,” “Gibberish/no comment,” “Unsure or undecided”* or “*Other”* (Table 5).

**Table 5:** Proportion (%) of open text responses from each treatment group when asked to provide further comments as to why, or why they would not, purchase eggs from hens housed in furnished cages/coops.

	**Control cage**	**Control coop**	**Educated cage**	**Educated coop**	**χ^2^**	***df***	***p***
Seems Good (%)	6.6^a^	7.4^a^	47.6^b^	38.4^b^	125.3	3	<0.001
Seems bad (%)	50.7^a^	17.4^b^	17.4^b^	14.5^b^	27.3^b^	3	<0.001
Welfare (%)	19.5^a^	12.1^a^	38.5^b^	29.8^b^	57.6	3	<0.001
Price (%)	26.0	20.9	31.6	21.4	1.38	3	0.71
Prefer Free-range (%)	30.2	30.2	21.9	17.7	7.7	3	0.05
Quality and taste (%)	28.4	17.6	29.7	24.3	1.3	3	0.73
Cage is a cage (%)	54.0^a^	6.3^b^	27.0^b^	12.7^b^	34.4	3	<0.001
I don't know (%)	45.5^a^	50.7^a^	0.7^b^	3.0^b^	150.8	3	<0.001
Other (%)	22.2	19.0	31.6	27.2	2.8	3	0.43
Not sure/undecided (%)	22.3	24.3	29.1	24.3	0.7	3	0.87
Gibberish/No comment (%)	26.4	29.2	20.8	23.6	3.6	3	0.31

*Participants were shown a control video using the term cage (control cage) or coop (control coop) or an educational video using the term cage (educated cage) or coop (educated coop). Differing subscript indicates a significant difference between treatment groups for each response (p < 0.05)*.

**Figure 4:**
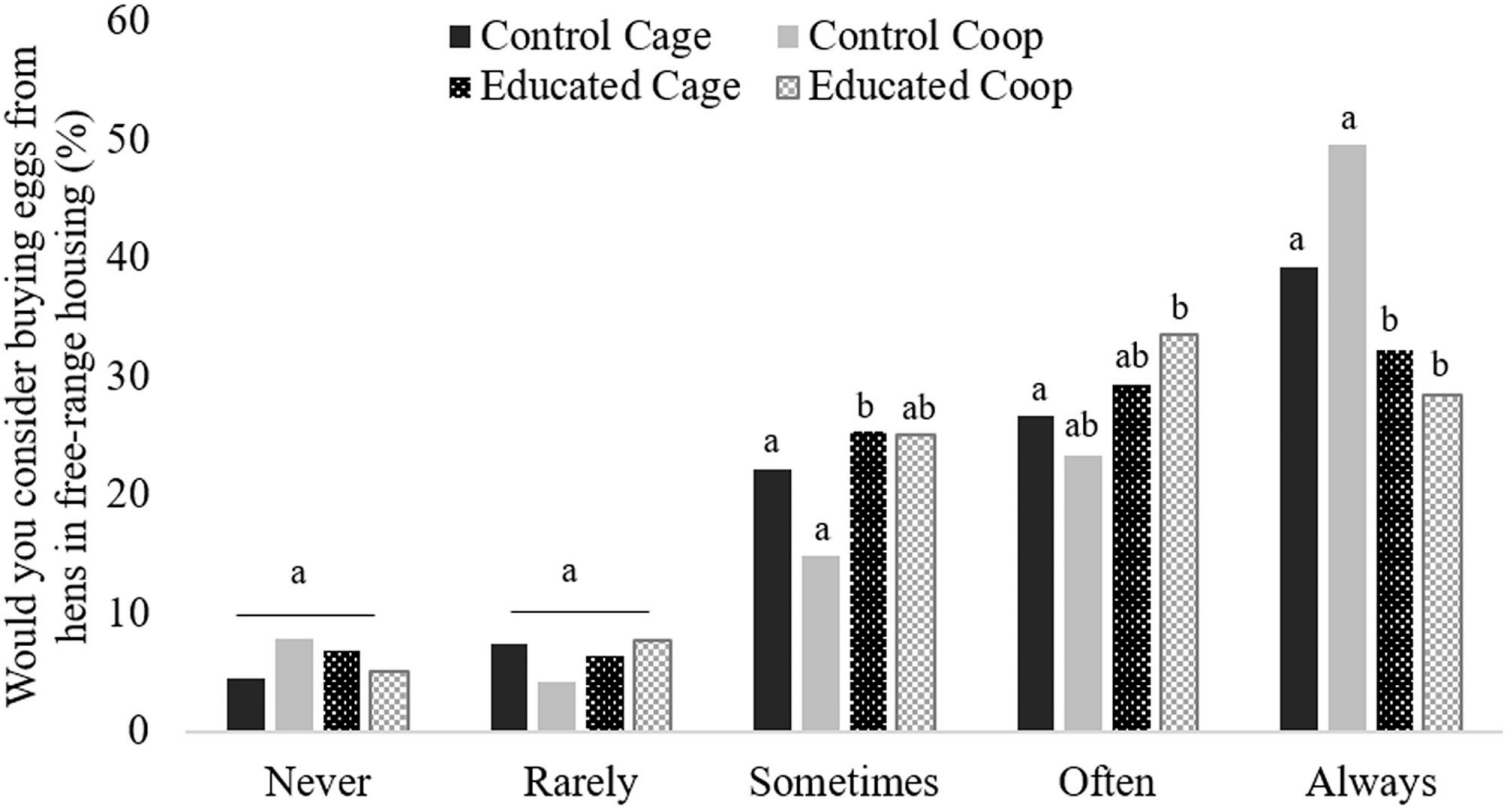
Frequency of response to the question “Which of the following hen housing systems would you consider buying from…free-range?” from participants after they were shown a control video using the term cage (control cage; solid dark gray bars) or coop (control coop; solid light gray bars) or an educational video using the term cage (educated cage; dark spotted bars) or coop (educated coop; gray checkered bars). Differing subscript indicates a significant difference between treatment groups for each response (*p* < 0.05).

Control groups were more likely to respond that they would *Always* purchase free-range eggs after the video intervention compared to educated groups. Control cage participants were less likely to respond *Often* than either educated group and educated groups were more likely to respond *Sometimes* than either control group (Figure 4). There was no difference in the level of support for conventional cage eggs, barn or aviary between the treatment groups.

## Discussion

This study examined the effect of rhetoric and education on willingness to support a novel housing system for laying hens. Specifically, we investigated if the inclusion of the term cage affected support for eggs from hens in different housing systems and whether education affected support for cage and other housing systems. Our results show that when respondents were given just the name independent of other information, their support for buying eggs from novel housing did not differ regardless of the terms used. However, when asked outright if they would support furnished systems, the control cage group were more likely to answer *No* than the control coop group. Education also improved support for furnished systems regardless of the language used and impacted the manner in which the housing system was discussed. We provide evidence that short educational videos may be required to increase support of a novel housing system in Australia, particularly if eggs are labeled with potentially loaded terminology, such as *cage*.

Control groups that were not educated were less likely to support eggs from a novel housing system when the term *cage* was used relative to *coop*, despite the two groups receiving the same video and questions. With no other factors differing between the groups, we are confident the reason for the difference lies in the rhetoric associated with the name. This is further evidenced by the significant number in control cage open-text responses themed “*a cage is a cage.”* Conversely, although the participants that were educated were more likely to support eggs from a novel housing system than control groups, there was no impact of language between the educated groups. Therefore, regardless of the name of the housing system, it was more likely to be supported by egg purchasers after viewing the educational video. This result is similar to Rohlf et al. (30) who reported an increase in support for furnished systems after an educational forum. For our study, both education groups were more likely to discuss the welfare implications of furnished systems in the open-text responses than either control group. This is possibly because of the welfare focus of the video. But this propensity to discuss housing in terms of welfare is positive, given the public's concern for animal welfare. The higher levels of doubt (i.e., those who responded *Maybe*) demonstrated by control coop participants compared to both educated groups and of control cage compared to educated cage were interesting. The fact that approximately the same percentage of control cage and control coops responded with *Maybe* as with *No* is evidence that the market need not dichotomize Cage vs. Cage Free, or Cage vs. Free Range [for instance (16)].

The increased support for furnished systems between control and education treatment groups is evidence that welfare-based education can improve support for novel housing systems. This result again demonstrates the impact education would have on consumer preferences for differently branded eggs. The nuance of a 5-point Likert scale suggests that the impact was most pronounced on the extreme ends of the scale, but an effect could be seen in the responses of *Rarely* and *Often* as well. Responses from *Rarely* to *Always* indicate there are at least some conditions under which participants would consider buying furnished eggs. After education, around a third of responders that initially responded *Never* did not change their answer. However, in both educated groups just over half of the participants changed from *Never* to either *Sometimes, Often* or *Always*, indicating that even those with hard stances on caged eggs can be influenced in the right circumstances. It is yet to be seen if other forms of education would work to influence support for furnished systems. For instance, quality, taste and other sensory attributes have been found to be strong motivators for purchasing free-range eggs (13). Furthermore, the non-consumers, those who responded that they would *Never* consider buying furnished eggs, should not be ignored. As demonstrated by Coleman et al. (5), it is not always the consumers who act as opinion leaders. Opinion leaders often perform more community behaviors, and regularly in opposition of livestock industries. This can include campaigns that actively dissuade consumers from purchasing certain eggs. If stakeholders wish to encourage adoption and meaningful change, it may be necessary to engage with both non-consumers and consumers even if they do not support furnished systems.

There was a significant difference between treatment groups in their responses when re-asked if they would purchase eggs from free-range chickens. This difference is best explained by the lower number of participants in the education groups willing to respond *Always* after the intervention video. This result suggests that once educated on the welfare pros and cons of free-range housing and/or, once free range is better defined in the participant's mind, they were less likely to limit themselves to purchasing free-range eggs and were more open to alternatives. It may also be interpreted that the additional choice of furnished eggs provides a sufficient alternative to free-range eggs. The analysis of individual choices and support change was beyond the scope of this study. This result is especially poignant given the low numbers of participants who could define free-range prior to the video intervention. There was no significant change in the support for conventional cages, despite the video presenting a balanced account of the welfare pros and cons of conventional caged systems. This is evidence that there might be more factors that influence levels of support for conventional cages than we can rightfully infer from this study. The welfare pros and cons of each housing system necessarily focus on different aspects of welfare that correspond with different values held by the respondents. If, didactic interventions are not often successful, as previously suggested (5, 36), it is more likely that the significant changes in free-range support occurred because the intervention clarified misconceptions or added to the participants' knowledge. Returning to the heuristics of egg production, Australians have been reported to consider chickens from free-range systems to be healthy, happy and not stressed (37). Similarly, Clemons and Day (38) found consumers were likely to define free-range as being the absence of cages (cage-free). Our video challenged these beliefs and backed them up with scientific evidence. However, the change of opinion was only with free-range eggs. There was no difference in the support for conventional cages. The reasons that support for free-range would change and not conventional cages should be further explored.

Within the coded text responses, the educated groups were more likely to state something positive about the furnished housing systems, while control groups were more likely to say something negative. It is worth noting, that unlike the multiple response survey choices, these two results are independent and do not represent two ends of a scale. Over half of those who responded negatively, (*Seems bad*) were from the control cage group. The reasons provided in this category vary but tend to comment negatively on the other themes i.e., “*I don't think cage eggs are safe, it is not a natural environment and they are not free to roam”* and “*Never heard of furnished cage so I won't buy them.”* These responses are similar to other qualitative research [see for instance, (13, 37, 38)].

Control cage participants were more likely to comment on the fact that the furnished system was a cage. Just over half of the respondents that stated “*a cage is a cage”* were from control cage and just under a third were from the educated cage group. Typical responses from control cage group included “*any caging is cruel”* and “*All cages are not good for chickens.”* Typical responses from educated cage group after being educated were “*Still caged.”* The use of *still* in the educated cage responses indicates the persistent belief that cages are not welfare friendly. The few remarks about cages by the *Coop* groups, demonstrates the power a rhetorical difference can have on the manner in which participants discuss egg choices. The differences between the control groups and educated groups suggest the heuristics associated with cages are based on fundamental misunderstandings of the caged-egg industry. Misunderstandings that can be rectified through education.

In our study, 72% of respondents from the Australian public that we sampled considered hen welfare as either *very important* or *extremely important*. Similar results have been reported by Coleman et al. (39) who found 72% of Victorians agreed with the statement *Farm animal welfare is an important concern*. However, the knowledge scores recorded in this survey were considerably lower than knowledge scores recorded in other studies. For instance, Coleman and Toukhsati (7) reported an approximate mean of 70% correct responses to questions based on industry practices and Rohlf et al. (30) reported a correct score of 85%. Both studies used multiple choice questions forcing participants to respond and offering the opportunity for guessing. Rohlf et al. (30) asked similar questions of their 20 participants, in their online forum. Although, as noted by the authors, their results were potentially influenced by recruiting via industry and animal welfare group sources. The questions in this study were not exceptionally difficult, especially as a correct response is necessary to justify many of the opinions held by the public. For example, only 11.5% could correctly define *free-range*. It is possible that the use of an open text response was not sufficient to elicit a correct response as participants were not given feedback on how much information was needed. If the question was asked in a different forum such as a focus group or online forum, participants might have been prompted to respond correctly. However, just 12.3% correctly answered the true/false question *Free-range flocks consist of* <*5,000 hens* as false prior to education interventions, so there is evidence that participants simply did not know the answer. One of the major differences between our study and that of Coleman and Toukhsati (7) is we had an additional option for the respondent to say “*I don't know.”* The additional answer category may have stopped people from guessing, thereby reducing the correct responses recorded by chance. On average, participants in our study responded with “*I don't know”* 50.8% of the time. This is important because it offers an alternative perspective on the common thinking around misconceptions. When forced to choose between only two options, people must express an opinion, and this does not necessarily mean it is a misconception. Our results demonstrate that people were, in general, more likely to say “*I don't know”* than to guess the correct answer. If this is mirrored in real world discussion, when asked about animal welfare and animal production, it is more likely people will respond with “*I don't know,”* or discussions of hearsay rather than speaking with authority. It remains to be seen just how much information someone needs before they feel they are an authority. It is also possible that the option of the “*I don't know”* response might have instilled some doubt in the respondent's mind, causing them to err on the side of caution.

When participants were asked to define “Free range,” three quarters of participants attempted a response and were incorrect. A little over 10 percent of people said they did not know the answer. The overall average of “*I don't know”* responses was just over half. The perceived knowledge of livestock industries and welfare is often greater than actual knowledge (6). The inability for the public to accurately define aspects of the Australian egg industry, and despite an overall concern for hen welfare may in part be explained by the paradigm known as the illusion of explanatory depth (IOED). IOED helps explain why people often overestimate the quality of their judgments and knowledge, especially when causal relations are complex [see Rozenblit and Keil (40); further explained below]. Furthermore, it may help explain why, despite information on hen housing being readily accessible, and despite the significant willingness to change support for hen housing systems once educated, the participant knowledge scores of our study were so low. The true effect of the IOED in the Australian public is unclear; however, it is important to know, because egg purchasing decisions are driven by more than financial constraints. The welfare of laying hens, and animals in general, is an important issue for the Australian public as was reiterated in our results. During the government's call for submissions to the open public consultation of the proposed draft Australian Animal Welfare Standards and Guidelines for Poultry, over 1,67,000 responses were received (41). Based on our study, these responses were potentially received from people without an accurate concept of the animal welfare consequences of standard poultry industry practices.

Even though we maintained an objective script and scientific approach, there were potential factors that might have influenced the outcomes. Firstly, highlighted by an information sheet preceding the survey, as mandated by the University of New England Ethics committee, this study was undertaken by scientists working at a university. It is currently unclear if the results would be the same if the survey was industry driven or supplied by an animal welfare organization. The appeal to authority that accompanies a University study likely added weight to our objectivity for some respondents, and potentially inspired distrust in others. The relative simplicity with which the support of Australians was changed opens the door for other authorities to educate using less-balanced and less-objective information while potentially receiving similar support changes in a direction desired by their organization.

Despite a representative sample of the Australian public, it pays to be cautious when reporting survey data. Self-reporting behavior is complicated, and people can be inaccurate when doing so (42). This is often because of the social desirability bias and reflects respondents' desire to appear prosocial. The social desirability bias causes survey respondents to make claims that are not accurately reflected by their behavior, often because the misrepresented behaviors are either valued or considered *good*—for the individual, their community, and/or society. If this is the case, it prompts the questions, which of the responses are not accurate and why are they considered more desirable? But perhaps more importantly, what is the prosocial impact of social desirability? For instance, if there is consumer support for novel housing systems, will the support be sustainable if the discourse surrounding the systems remains negative?

## Conclusion

We hypothesized that the rhetoric used in labeling eggs affects consumers' preferences. However, we found no evidence that changing the name of a novel housing system changed support for those eggs. There was evidence that the name of the housing system, in isolation from any educational intervention, could change the manner in which it is discussed. It is not clear how this might affect support for novel housing systems in the future. We further hypothesize that consumers' preferences for eggs from these housing systems will change when they are made aware of scientific knowledge about housing systems. Our study demonstrated that participants were relatively unaware of industry practices. Once educated we saw a significant decrease in support for free-range eggs and an increase in support for furnished housing although the name of the furnished system did not affect participant support. Finally, we hypothesized that watching an educational video on the egg industry would change the way people discuss eggs. It is evident from our study that the themes discussed post-video differed from the treatment groups that were not shown an educational video. Participants were more positive about furnished housing and more open to discussions of animal welfare.

If the Australian egg industry wishes for the Australian public to adopt and support furnished cages, educational videos involving objective scientific evidence about welfare is meaningful step forward. Any reluctance to support furnished cages appears to be from a lack of knowledge around what furnished cages are, and not because of the name.

## Data Availability Statement

The raw data supporting the conclusions of this article will be made available by the authors, without undue reservation.

## Ethics Statement

This study was approved by the University of New England Human Ethics Committee (HE18–284). The patients/participants provided their written informed consent to participate in this study.

## Author Contributions

HN and PT wrote the manuscript and conducted all analysis. All authors helped with developing the concepts, methodologies, conceiving the experiment and contributed to manuscript writing. All authors contributed to the article and approved the submitted version.

## Funding

This work was funded by Poultry Hub Australia (grant number 18-429).

## Conflict of Interest

The authors declare that the research was conducted in the absence of any commercial or financial relationships that could be construed as a potential conflict of interest.

## Publisher's Note

All claims expressed in this article are solely those of the authors and do not necessarily represent those of their affiliated organizations, or those of the publisher, the editors and the reviewers. Any product that may be evaluated in this article, or claim that may be made by its manufacturer, is not guaranteed or endorsed by the publisher.
