# Supplementary material for: A Cage Is a Cage, Unless You Educate. Rhetoric Negatively Impacts Support for a Novel Housing System for Laying Hens Unless the Public Are Educated
Source: Front Vet Sci. 2022 Feb 18;9:797911. doi: 10.3389/fvets.2022.797911 (PMC8894605; doi:10.3389/fvets.2022.797911)
Supplement: Supplementary file 1 [file Data_Sheet_1.docx]

Supplementary Material

# Script for video interventions

Bold blue text indicates areas tested pre- and post- intervention.

## Control video intervention script

Meet the domesticated hen. Hens are descendants of Jungle Fowl that inhabited dense jungle habitats throughout Asia (1). Jungle fowl were domesticated more than 7000 years ago (2) and were originally kept and bred for cock fighting and ceremonial purposes. The red jungle fowl can still be found in the wild in countries such as India, Burma and Thailand, but the domesticated hen can be found all over the world. There are different terms to refer to chickens depending on their age and stage of life. A chick is newly hatched and typically covered in soft fluff. When fluff is replaced with feathers around 6 weeks of age, but still sexually immature we call female’s pullets and males cockerels. After sexual maturity, females are called hens and males roosters. If males are castrated, he is referred to as a capon. Hens do not need roosters to produce an egg. But if a rooster does mate with a hen, she produces a fertilised eggs (not the ones typically sold and eaten in Australia). These fertilised eggs grow chicks in 20-21 days (3). Chicks communicate with their mother whilst in the egg, displaying a series of vocalisations to which mother hens respond to accordingly (4). After hatching, chicks stay close to the mother hen for protection and to gain some valuable lessons, such as what is good to eat and what is potentially harmful (5). After a relatively short period of time, chicks will become less reliant on their mother and begin to explore the world and meet new chickens. The first encounter between two unfamiliar adult chickens will likely result in aggression. This is to determine a social structure of the group and so they both know who is higher in the pecking order (6). When these chickens meet again later in life they remember their relative rank. The more dominate hen will assert her dominance by threats and specific postures to avoid any further aggression. Chickens can recognise up to 100 individuals and their social status within that group. Vocalisations are an important communication tool for chickens. There have been around 30 different vocalisations described although we still don’t know what many of them mean (7). Roosters use vocalisations to alert hens to a nice bit of food. Roosters will vocalise whilst picking up and dropping food particles, this is called ‘tid-bitting’ a behaviour he uses to attract a mate (8). Subordinate roosters also want find a mate, so likewise will perform tid-bitting but without the vocalisations to avoid attracting attention from a dominate rooster (9). Nowadays, there are more chickens in the world than any other species of bird. Chickens are kept for food, as companions and therapy. The chickens are the closest living relative of dinosaurs, with hundreds of different chicken breeds, they out number humans nearly 3 to 1. So next time someone calls you chicken, think of the evolutionary success and simply reply ‘thank you’

Video link: https://youtu.be/deHHEaDeaVQ

## Furnished cage video intervention script

Meet the laying hen, **she can produce over 300 eggs each year**, and in Australia she may be housed in a conventional cage or free-range housing system. In regards to hen welfare, both housing systems have pro’s and con’s. Conventional cage housing is where hens are kept inside a barn, in multitiered cages with wire mesh floors which prevents them from being in direct contact with their faeces. Hens are housed in groups, of more natural group size than in non-caged systems. This system improves the prevention of some diseases. However, the conventional cage restricts the expression of some behaviors such as wing flapping, dustbathing, perching, nesting and scratching. At the end of their production life hens are more likely to have osteoporosis and bone fractures (10). In free-range housing, hens have regular access to an outdoor range during the day and kept inside a shed overnight. Hens can express a broader range of behaviors in the shed and on the range than in caged systems. However, access to the outdoors increases the risk of parasites, disease (including exposure to avian influenza), and predation by foxes and eagles. Also, hens are more likely to collide with object and each other which can cause injuries. Free-range systems typically house large flocks of between **1000 – 20,000 hens (11),** such large group sizes can increase the incidence of severe feather pecking and cannibalism. **To minimize the damage caused by severe feather pecking, the tip of the beak of day-old chicks is often removed in a process known as beak trimming.** As you can see there are **challenges to hen welfare in both conventional cage and free-range housing systems.** As a result, industry and scientists have been working to find alternatives. Introducing the Furnished Cage! A furnished cage is a housing system that keeps hens in relatively small flock sizes in a cage that contains a perch, nest box and a scratchpad to increase the expression of specific behaviors that they can’t express as well in conventional cages; such as roosting at night (which can improve bone strength (12)), nesting behavior and foraging. In furnished cage housing, hens are kept off their faeces and are not at risk of diseases associated with outdoor ranges. Consequently hens from Furnished cages are often in better health (13) and are less likely to die than in free-range systems (mortality 3% FC compared to FR 22% (14)). Although scientists don’t know the exact perfect design for FC, this alternative system reduces the welfare compromises of more traditional hen housing systems. Such that the health of the hens are improved relative to free-range housing, and the ability to perform motivated behavior is permitted unlike conventional cages. So, would you support the development of the furnished cage housing system in Australia?

Video link: https://youtu.be/xJIzo6Q7W5c

## Furnished coop video intervention script

Meet the laying hen, **she can produce over 300 eggs each year**, and in Australia she may be housed in a conventional cage or free-range housing system. In regards to hen welfare, both housing systems have pro’s and cons. Conventional cage housing is where hens are kept inside a barn, in multitiered cages with wire mesh floors which prevents them from being in direct contact with their faeces. Hens are housed in groups of 9-10 hens, is a more natural group size than in non-caged systems. This system improves the prevention of some diseases. However, the conventional cage restricts the expression of some behaviors. At the end of their production life hens are more likely to have osteoporosis and bone fractures.(10). In free-range housing, hens have regular access to an outdoor range during the day and kept inside a shed overnight. Hens can express a broader range of behaviors in the shed and on the range than in caged systems, behaviors such as foraging, wing flapping, dustbathing, perching, nesting and scratching. However, access to the outdoors increases the risk of disease (including exposure to avian influenza), parasites, and predation by foxes and eagles. Also, hens are more likely to suffer physical injuries such as bone breakages. Free-range systems typically house large flocks of between **1000 – 20,000 hens (11),** such large group sizes can increase the incidence of severe feather pecking and cannibalism. **To minimize the damage caused by severe feather pecking, the tip of the beak of day-old chicks is often removed in a process known as debeaking.** As you can see there are **challenges to hen welfare in both conventional cage and free-range housing systems.** As a result, industry and scientists have been working to find alternatives. Introducing the Furnished Coop! A furnished coop is a housing system that keeps hens in relatively small flock sizes in a cage that contains a perch, nest box and a scratchpad to increase the expression of specific behaviors that they can’t express as well in conventional cages; such as roosting at night (which can improve bone strength (12)), nesting behavior and foraging. In furnished cage housing, hens are kept off their faeces and are not at risk of diseases associated with outdoor ranges. Consequently hens from Furnished coops are often in better health (13) and are less likely to die than in free-range systems (mortality 3% FC compared to FR 22% (14)). Although scientists don’t know the exact perfect design for furnished coops, this alternative system reduces the welfare compromises of more traditional hen housing systems. Such that the health of the hens are improved relative to free-range housing, and the ability to perform motivated behavior is permitted unlike conventional cages. So, would you support the development of the furnished coop housing system in Australia?

Video link: <https://youtu.be/CjPCxTYyvuY>

1. Liu Y-P, Wu G-S, Yao Y-G, Miao Y-W, Luikart G, Baig M, et al. Multiple maternal origins of chickens: out of the Asian jungles. Molecular phylogenetics and evolution. 2006;38(1):12-9.

2. Storey AA, Athens JS, Bryant D, Carson M, Emery K, Higham C, et al. Investigating the global dispersal of chickens in prehistory using ancient mitochondrial DNA signatures. Plos One. 2012;7(7):e39171.

3. Hamburger V, Hamilton HL. A series of normal stages in the development of the chick embryo. Developmental dynamics. 1992;195(4):231-72.

4. Tuculescu RA, Griswold JG. Prehatching interactions in domestic chickens. Anim Behav. 1983;31(1):1-10.

5. Nicol C. How animals learn from each other. Appl Anim Behav Sci. 2006;100(1-2):58-63.

6. Dawkins MS. Distance and social recognition in hens: implications for the use of photographs as social stimuli. Behaviour. 1996;133(9):663-80.

7. Collias N, Joos M. The spectrographic analysis of sound signals of the domestic fowl. Behaviour. 1953;5(1):175-88.

8. Wood-Gush DGM. The behaviour of the domestic fowl1971.

9. Smith CL, Taylor A, Evans CS. Tactical multimodal signalling in birds: facultative variation in signal modality reveals sensitivity to social costs. Anim Behav. 2011;82(3):521-7.

10.Lay D, Fulton R, Hester P, Karcher D, Kjaer J, Mench J, et al. Hen welfare in different housing systems. Poultry Sci. 2011;90(1):278-94.

11.Singh M, Ruhnke I, de Koning C, Drake K, Skerman AG, Hinch GN, et al. Demographics and practices of semi-intensive free-range farming systems in Australia with an outdoor stocking density of≤ 1500 hens/hectare. Plos One. 2017;12(10):e0187057.

12.Barnett J, Tauson R, Downing J, Janardhana V, Lowenthal J, Butler K, et al. The effects of a perch, dust bath, and nest box, either alone or in combination as used in furnished cages, on the welfare of laying hens. Poultry Sci. 2009;88(3):456-70.

13.Tauson R. Management and housing systems for layers–effects on welfare and production. World's Poultry Science Journal. 2005;61(3):477-90.

14.Elson H. Poultry welfare in intensive and extensive production systems. World's Poultry Science Journal. 2015;71(3):449-60.

15.Widowski TM, Classen H, Newberry R, Petrik M, Schwean-Lardner K. Code of Practice for the Care and Handling of Pullets, Layers, and Spent Fowl: Poultry (Layers): Review of Scientific Research on Priority Issues: University of Saskatchewan; 2013.

# Survey

1. Please select the age group you fall under (Under 18 years; 18-24 years old; 25-34 years old; 35-44 years old; 45-54 years old; 55-65 years old; 65+ years old)
2. Gender (Male; Female; Other (please explain, if you want to)___)
3. What state/territory are you from? (New South Wales; Victoria; Queensland; South Australia; Western Australia; Tasmania; Australian Capital Territory; Northern Territory)
4. Do you primarily live in an Australian capital city? (Yes; No)
5. What is your current postcode?
6. Highest level of education completed: (Less than a high school diploma; High school diploma; Associate degree (e.g. diploma); Bachelor's degree; Master's Degree; Professional degree (MD, DVM); Doctorate (PhD); I don't wish to answer)
7. Annual household income: (Less than $20,000; $20,000-$39,999; $40,000-$69,999; $70,000-$99.999; $100,000-$150,000; Over $150,000)
8. Diet type: (Variety of food, including white and red meat; Vegetarian, no meat consumption (including no fish consumption), but may consume dairy or eggs; Entirely plant based, no animal products at all; Other (please explain) )
9. Are you a member of an animal welfare, or animal rights oraganisation (for example RSPCA, PETA)? (No; Yes: please name the organisation(s); I previously was but am not anymore: please name the organisation(s))
10. How often do you consume eggs in an average week? (Never; Once per week; 2-3 times per week; More than 3 times per week)
11. Why don't you eat eggs?
12. How important are the following factors in your decision not to eat eggs? (Unimportant; Slightly important; Moderately important; Important; Very Important)
    1. Animal Welfare
    2. Environmental Impact
    3. Cost
    4. I don't like the taste
    5. Other (please explain)
13. Where do you (mostly) buy your eggs? (I don't buy eggs (Why not?) ___; Supermarket; Farmers markets; Organic stores only; Other (explain) ____)
14. Which of the following hen housing systems would you consider buying from?
15. Which of the following hen housing systems would you consider buying from? (Never; Rarely; Sometimes; Often; Always; I am unfamiliar with this housing system)
    1. Conventional Cage
    2. Free-range
    3. Barn
    4. Furnished Cage
    5. Aviary
16. Rank the following factors that contribute to your decision when purchasing eggs
    1. Price
    2. Environmental sustainability
    3. Food safety
    4. Locally produced
    5. Housing system
17. How do you define Animal Welfare?
18. Indicate your level of agreement with the following descriptions of good animal welfare (Strongly disagree; Disagree; Neither agree nor disagree; Agree; Strongly agree)
    1. No disease or injury
    2. Excellent growth and production
    3. Expression of natural behaviours
    4. Good mental health
    5. Being alive
    6. No / minimal stress
    7. Freedom
19. Which of the following are important for good hen welfare? (Not at all important; Slightly important; Moderately important; Very important; Extremely important)
    1. Access to food and water
    2. Protection from disease and predators
    3. Access to natural resources
    4. Feeling good
    5. Space
    6. Sunlight
    7. Choice
20. I think the welfare of Australian commercial hens is: (Very bad; Bad; OK, but room for improvement; Adequate; Good; Excellent)
21. Hen welfare is: (Not at all important; Slightly important; Moderately important; Very important; Extremely important)
22. The welfare of laying hens is not an important consideration to my shopping choices (Strongly disagree; Disagree; Neither agree nor disagree; Agree; Strongly agree)
23. I should make the effort to buy eggs that are produced with good hen welfare practices(Strongly disagree; Disagree; Neither agree nor disagree; Agree; Strongly agree)
24. I think it is important to lobby governments to improve the welfare of laying hens(Strongly disagree; Disagree; Neither agree nor disagree; Agree; Strongly agree)
25. The welfare of laying hens is something that my partner/family would expect me to consider when making egg shopping choices (Strongly disagree; Disagree; Neither agree nor disagree; Agree; Strongly agree)
26. My partner/family would expect me make the effort to buy eggs that are produced with good animal (Strongly disagree; Disagree; Neither agree nor disagree; Agree; Strongly agree)
27. My partner/family expects me to lobby governments to improve the welfare of laying hens (Strongly disagree; Disagree; Neither agree nor disagree; Agree; Strongly agree)
28. It is easy to take in to consideration hen welfare when making egg shopping choices (Strongly disagree; Disagree; Neither agree nor disagree; Agree; Strongly agree)
29. I find it takes too much effort to buy eggs that are produced with good animal welfare practices (Strongly disagree; Disagree; Neither agree nor disagree; Agree; Strongly agree)
30. I would find it too difficult to lobby the government to improve the welfare of laying hens (Strongly disagree; Disagree; Neither agree nor disagree; Agree; Strongly agree)
31. How informed you are on the following matters? (I don't know anything; I know a little bit; I know as much as anyone else; I know more than the average person; I consider myself an expert on the topic)
    1. Animal Welfare
    2. Laying hen welfare
    3. The Australian Laying hen industry
    4. Management practices of laying hens
    5. Animal welfare legislation
32. Where do you obtain information about laying hens from? (Never; Sometimes; Most of the time)
    1. Radio
    2. Internet news sites
    3. Social networking sites (e.g. Facebook)
    4. Television
    5. Print media
    6. Animal welfare organisations (e.g. RSPCA)
    7. Supermarkets
    8. Industry bodies
    9. Other (please explain)
33. How much do you trust the information from the following sources (Complete distrust; Neither trust nor distrust; Some distrust; Some trust; Complete trust)
    1. Radio
    2. Internet news sites
    3. Social networking sites (e.g. Facebook)
    4. Television
    5. Print media
    6. Animal welfare organisations (e.g. RSPCA)
    7. Supermarkets
    8. Industry bodies
    9. Other (please explain)
34. Poultry knowledge (True; False; I don’t know)
    1. Shed lights are on 24 hours a day so each hen produces two eggs every day
    2. Yolk colour is related to housing system
    3. Free-range flocks consist of less than 5,000 hens
    4. Hens are killed between 16 - 25 weeks of age because their egg production decreases
    5. Chicken meat and eggs come from two different types of chickens
    6. Commercial strains of hens each produce over 300 eggs per year
    7. The current outdoor range stocking density for hens in free-ranged egg production systems is 10,000 hens/hectare
    8. Moulting is practiced in Australia
    9. Hens in free-range housing systems have no welfare problems
35. Define the following terms: please write 'Unknown' if you don't know
    1. Free-range
    2. Beak-trimming
    3. Moulting
    4. Feed conversion ratio
36. [Video Intervention ]

Please copy/paste the below address into a new browser window and watch the short video: You will be able to return to this browser and continue the survey once the video is complete

Thank you for watching our video. We will now be asking similar questions as before- don’t worry, the survey hasn’t gone backwards.

1. Poultry knowledge (True; False; I don’t know)
   1. Shed lights are on 24 hours a day so each hen produces two eggs every day
   2. Yolk colour is related to housing system
   3. Free-range flocks consist of less than 5,000 hens
   4. Hens are killed between 16 - 25 weeks of age because their egg production decreases
   5. Chicken meat and eggs come from two different types of chickens
   6. Commercial strains of hens each produce over 300 eggs per year
   7. The current outdoor range stocking density for hens in free-ranged egg production systems is 10,000 hens/hectare
   8. Moulting is practiced in Australia
   9. Hens in free-range housing systems have no welfare problems
2. Define the following terms: please write 'Unknown' if you don't know
   1. Free-range
   2. Beak-trimming
   3. Moulting
   4. Feed conversion ratio
3. How do you define Animal Welfare?
4. Indicate your level of agreement with the following descriptions of good animal welfare (Strongly disagree; Disagree; Neither agree nor disagree; Agree; Strongly agree)
   1. No disease or injury
   2. Excellent growth and production
   3. Expression of natural behaviours
   4. Good mental health
   5. Being alive
   6. No / minimal stress
   7. Freedom
5. Which of the following are important for good hen welfare? (Not at all important; Slightly important; Moderately important; Very important; Extremely important)
   1. Access to food and water
   2. Protection from disease and predators
   3. Access to natural resources
   4. Feeling good
   5. Space
   6. Sunlight
   7. Choice
6. I think the welfare of Australian commercial hens is: (Very bad; Bad; OK, but room for improvement; Adequate; Good; Excellent)
7. Hen welfare is: (Not at all important; Slightly important; Moderately important; Very important; Extremely important)
8. Would you consider purchasing furnished cage/coop eggs (Yes; No; Maybe; Yes, but dependent on___)
9. Please provide further comment on your answer above (e.g. why, why not, only if)
10. Which of the following hen housing systems would you consider buying from? (Never; Rarely; Sometimes; Often; Always; I am unfamiliar with this housing system)
    1. Conventional Cage
    2. Free-range
    3. Barn
    4. Furnished Cage
    5. Aviary
11. Is there anything you would like to contribute to research on laying hen welfare? Any comments or concerns? Remember, this survey is anonymous.
